# Microbiota in anorexia nervosa: The triangle between bacterial species, metabolites and psychological tests

**DOI:** 10.1371/journal.pone.0179739

**Published:** 2017-06-21

**Authors:** Francesca Borgo, Alessandra Riva, Alberto Benetti, Maria Cristina Casiraghi, Sara Bertelli, Stefania Garbossa, Simona Anselmetti, Silvio Scarone, Antonio E. Pontiroli, Giulia Morace, Elisa Borghi

**Affiliations:** 1 Department of Health Sciences, Università degli Studi di Milano, Milan, Italy; 2 ASST Santi Paolo e Carlo, Milan, Italy; 3 Department of Food, Environmental and Nutritional Sciences, Università degli Studi di Milano, Milan, Italy; Instituto de Agroquimica y Tecnologia de Alimentos, SPAIN

## Abstract

Anorexia nervosa (AN) is a psychiatric disease with devastating physical consequences, with a pathophysiological mechanism still to be elucidated. Metagenomic studies on anorexia nervosa have revealed profound gut microbiome perturbations as a possible environmental factor involved in the disease. In this study we performed a comprehensive analysis integrating data on gut microbiota with clinical, anthropometric and psychological traits to gain new insight in the pathophysiology of AN. Fifteen AN women were compared with fifteen age-, sex- and ethnicity-matched healthy controls. AN diet was characterized by a significant lower energy intake, but macronutrient analysis highlighted a restriction only in fats and carbohydrates consumption. Next generation sequencing showed that AN intestinal microbiota was significantly affected at every taxonomic level, showing a significant increase of *Enterobacteriaceae*, and of the archeon *Methanobrevibacter smithii* compared with healthy controls. On the contrary, the genera *Roseburia*, *Ruminococcus* and *Clostridium*, were depleted, in line with the observed reduction in AN of total short chain fatty acids, butyrate, and propionate. Butyrate concentrations inversely correlated with anxiety levels, whereas propionate directly correlated with insulin levels and with the relative abundance of *Roseburia inulinivorans*, a known propionate producer. BMI represented the best predictive value for gut dysbiosis and metabolic alterations, showing a negative correlation with *Bacteroides uniformis* (microbiota), with alanine aminotransferase (liver function), and with psychopathological scores (obsession-compulsion, anxiety, and depression), and a positive correlation with white blood cells count. In conclusion, our findings corroborate the hypothesis that the gut dysbiosis could take part in the AN neurobiology, in particular in sustaining the persistence of alterations that eventually result in relapses after renourishment and psychological therapy, but causality still needs to be proven.

## Introduction

Eating disorders are an important cause of physical and psychosocial morbidity in adolescent girls and adult women [1]. Anorexia nervosa (AN) is a life threatening mental illness characterized by extreme energy intake restriction that leads to dangerous low body weight, associated with gastrointestinal, cardiovascular, immunological diseases and metabolic abnormalities [2,3]. AN has an estimated incidence of 8/100,000 subjects per year [4], and its mortality is one of the highest in psychiatric disorders [5]. Despite its devastating physical consequences and strong social impact, its pathophysiology remains unclear [1]. AN patients have a deceptive self-image, and several studies have shown that the onset of anxiety disorder precedes the onset of AN pathology in most cases [6,7]. The correlation between anorexia and anxiety disorders is still to be elucidated.

Animal studies have greatly improved the understanding of AN molecular basis. Particularly, the use of *anx*/*anx* mouse strain generated new theories on hypothalamic degeneration and inflammation that might explain some of the behavioral characteristics of AN [8]. On the other hand, molecular studies on AN are also providing insight into how diet and nutrition can alter gene expression [9], which may in turn increases the risk for AN.

Gut microbiota has been demonstrated to play a role in different metabolic functions such as regulation of weight gain, energy harvest from diet and insulin secretion [10], and to be highly influenced by diet and life style [11]. Some studies have demonstrated a different microbial composition between obese and normal weight subjects [12,13], but very little has been explored in patients affected by AN. In addition, recent findings established the presence of a gut-brain axis [14], and alterations in microbiota composition have been linked to anxiety and depression [15], frequent in AN patients [16–18]. Indeed, a vicious cycle between microbiota and psychopathological traits has been suggested, with microbiota alteration participating in hypersensitivity of the hypothalamus-pituitary-adrenal (HPA) axis in response to stressors [19], and psychological stress promoting microbial translocation that boosts gut inflammation [20].

The microbiota produces an array of bioactive metabolic products capable of entering systemic circulation. These metabolic products can have profound effects on host metabolism, immune function, and gene expression in many organ systems, including the central nervous system (CNS) [21]. Short chain fatty acids (SCFAs), the main microbial metabolites, are volatile fatty acids produced by bacteria in the large bowel as fermentation products from food components undigested/unabsorbed in the small intestine. Acetic acid, propionic acid, and butyric acid are the most abundant, representing 90–95% of SCFAs in colon (ratio of 3:1:1) [22]. Only a small percentage, about 5%, of SCFAs produced in the colon can be recovered in feces; butyrate is rapidly used as an energy source for colonocytes, whereas most of acetate and propionate enter the portal circulation [23]. Butyrate and to a lesser extent propionate and acetate are histone deacetylase (HDAC) inhibitors with potential effects on gene expression in human cells [24]. Propionate crosses the blood brain barrier and enters the CNS [25] and, like butyrate, affects various physiological processes such as cell signaling, neurotransmitter synthesis and release, free radical production, and mitochondrial function. From a metabolic point of view, propionate is the preferred precursor by liver for cholesterol synthesis regulation and gluconeogenesis[26]. Acetate is the main SCFA in blood, and plays a key metabolic role in peripheral tissues acting as a substrate for lipogenesis [26].

Data available on microbiota in AN population suggested differences in the microbial ecology compared with normal weight individuals. Pfleiderer and colleagues discovered 11 new bacterial species belonging to *Firmicutes*, *Bacteroidetes* and *Actinobacteria* phyla in AN patients admitted in the hospital [27], whereas other researchers found an increased abundance of the archeon *Methanobrevibacter smithii* [28]. A lower bacterial diversity characterized AN patients with greater depression and anxiety [6], and changes in microbial composition and SCFA profiles were linked to several gastrointestinal symptoms [29].

Up to date, patients’ management is based on psychotherapy and drug administration to reduce anxiety or depression but a treatment that targets the causal factors of anorexia nervosa is still lacking. Due to the spreading of this serious disease, any potential treatment needs to be carefully considered.

In this context, our study aimed at integrating microbiome data with clinical and psychological traits in order to elucidate the possible relationship between nutritional status, and the microbiota-gut-brain axis in Anorexia Nervosa. Indeed, unraveling this link could pave the way to develop alternative approaches that modulate intestinal microbiota (e.g. probiotics, prebiotics), affecting those physiological pathways that are known to be altered in AN.

## Materials and methods

### Study population and design

We enrolled patients with diagnosis of Anorexia nervosa (AN) referred to the Eating Disorder Unit of ASST Santi Paolo e Carlo in Milan from January 2016 to June 2016. The study was reviewed and approved by the local medical ethics committee (Comitato Etico Interaziendale Milano Area A—protocol number 2015/ST/122). Written informed consent was given by all enrolled subjects or parent. Inclusion criteria were: a diagnosis of anorexia according to *Diagnostic and Statistical Manual of Mental Disorders*, *Fifth Edition* criteria [30], female sex, restrictive behavior and very low body mass index (BMI, <14 kg/m^2^) at enrollment. Control group (CTR), matched for age and sex, was enrolled on a voluntary basis among hospital personnel and hospital staff’s relatives. Exclusion criteria were: use of antibiotics or probiotics in the month before the enrolment, celiac disease, irritable bowel syndrome, history of colorectal cancer, diabetes mellitus, binge eating or purging behavior, recent enteral/parenteral nutrition.

To avoid biased psychological tests, none of the participants was aware of hypotheses, aims or the clinical protocol of the study.

### Sample collection

Stool samples were collected from all enrolled subjects and stored at -80°C until use. Blood samples were taken in the morning under fasting conditions and routinely tested for: cell blood count, glucose, insulin, cholesterol (total, low density, and high density lipoprotein cholesterol), triglycerides, aspartate aminotransferase (AST), alanine aminotransferase (ALT), glutamyltransferase (GGT), alkaline phosphatase (AP), cholinesterase (CHE), sodium, potassium, total bilirubin, pancreatic lipase, pre-albumin, albumin, transferrin, ferritin, iron binding capacity (TIBC), indices of renal functions (creatinine, urea), total immunoglobulin A, transglutaminase antibodies, thyroid stimulating hormone level. During blood drawing an additional sample was collected, processed to obtain serum sample, and subsequently stored at -80°C for metabolomic tests.

### Dietary evaluation and anthropometric data

Each participant in the study filled in a three-day food record. Food diaries were processed by dietitians to calculate the average amount of energy and nutrient intake, according to food composition tables obtained by the *Italian aliments composition database for epidemiological studies* (http://www.bda-ieo.it).

From all subjects we collected anthropometric data: weight, height and body mass index (BMI) at the time of enrolment. BMI was calculated as ratio between weight, expressed in kilograms, and height, expressed in meters squared [31]. Moreover, each participant’s body composition was estimated using bioelectrical impedance analyses (BIA) in order to obtain the following parameters: fat body mass, fat-free body mass, bone mass, total body water [32].

### Psychopathology assessment

The severity of eating disorder and psychopathology tests were assessed by means of the Symptom Checklist-90 (general psychopathology, [33]), Eating Disorder Inventory 2 (eating disorder, [34]), State Trait Anxiety Inventory (anxiety disorder, [35]) and Beck Depression Inventory [depressive symptoms, [36]). Psychologists and psychiatrists (all extensively trained in the use of the instruments) conducted clinical evaluations.

The Eating Disorder Inventory-2 (EDI-2) is a reliable self-reported questionnaire that explores typical cognitive and behavioral characteristics of eating disorders. A total of 91 items, answered on a 6-point Likert scale, provide standardized scores on 11 subscales: drive for thinness, body dissatisfaction, bulimia, effectiveness, perfectionism, interpersonal distrust, interoceptive awareness, maturity fears, ascetism, impulse regulation, and social insecurity. The EDI-2 total score (sum of scores in each scale) was used to examine overall eating disorder severity.

The Symptoms Checklist-90 revised (SCL90) is a self-reported measure used to assess general psychopathology. The 90-items questionnaire allows extracting nine primary psychopathological symptom dimensions: (1) somatization; (2) obsession-compulsion; (3) interpersonal sensitivity; (4) depression; (5) anxiety; (6) hostility; (7) phobic anxiety; (8) paranoid ideation; and (9) psychoticism. A global index is also present to evaluate the Global Severity Index (GSI).

The State Trait Anxiety Inventory Scale (STAI, form Y) is composed by two subscales (20 items each): the State Anxiety Scale (STAI-1), and the Trait Anxiety Scale (STAI-2). STAI-1 scale evaluates the current anxiety status, asking how respondent feel “right now”, using items that measure subjective feelings of apprehension, tension, nervousness, worry, and activation/arousal of autonomic nervous system. The STAI-2 scale evaluates relatively stable aspects of “anxiety proneness”, including general states of calmness, confidence, and security.

Beck Depression Inventory-II (BDI-II) is one of the most commonly used self-reported measure for major depressive disorders [36,37]. The questionnaire is based on 21 items, divided on 2 subscales: affective and somatic. Each item is rated on a 4-point scale ranging from 0 to 3. The maximum total score is 63. Total score is used to estimate the level of depression: 0–13, minimal depression; 14–19, mild depression; 20–28, moderate depression; 29–63, severe depression.

### DNA extraction and sequencing

Total bacterial DNA extraction was performed using the Spin stool DNA kit (Stratec Molecular, Berlin, Germany), according to the manufacturer’s instructions and amplified by PCR. 25 ng of DNA extracted from each stool sample was utilized to construct a sequencing library. 16S rRNA gene amplicon libraries were performed with a two-step barcoding approach according to Illumina 16S Metagenomic Sequencing Library Preparation (www.illumina.com). In the first-step PCR, 16S rRNA genes of all bacteria were amplified as described by Klindworth et al. [38]. For library preparation, DNA samples coming from first PCR step were amplified with dual-index primers using Nextera DNA Library Preparation Kit (Illumina, San Diego, CA, USA). Each sample possessed specific barcode sequences at the front and end of the PCR amplicon to discriminate among each other in the pooled library. Both library concentration and exact product size were measured using a KAPA Library Quantification Kit (Kapa Biosystems, Woburn, MA, USA) and an Agilent 2100 Bioanalyzer System (Agilent, Santa Clara, CA, USA), respectively.

A pooled library (20 nM) and a PhiX control v3 (20 nM) (Illumina) were mixed with 0.2 N fresh NaOH and HT1 buffer (Illumina) to produce the final concentration at 12 pM each. The resulting library was mixed with the PhiX control v3 (5%, v/v) (Illumina) and 600 μL loaded on a MiSeq® v2 (500 cycle) Reagent cartridge for sequencing. All sequencing procedures were monitored through the Illumina BaseSpace® website. Detection of sequencing fragments was performed on Illumina MiSeq platform with a 250PE protocol. Samples were run in pool of up to 48 plex to obtain an average of 0.15 GBases of sequence data per sample. FastQ files were generated at the end of the run to perform the quality control. Sequencing reads are available in NCBI Short Read Archive (SRA, http://www.ncbi.nlm.nih.gov/sra) under ID PRJNA375065.

### Real-time PCR for *Methanobrevibcater smithii* quantification

Absolute quantification by Real-time PCR was performed using *M*. *smithii* DSM-861 as control strains (DSM: Deutsche Sammlung von Mikroorganismen und Zellkulturen). Firstly, microbial DNA was extracted using Prepman Ultra (Applied Biosystem, USA). Real Time PCR was carried out using the StepOne Plus instrument (Applied Biosystems) and the SYBR® Green chemistry (ThermoScientific, USA). The analysis was performed in a total volume of 15 μl, and each sample analyzed in triplicate. Standard curve was carried out using five serial dilutions of control DNA and specific 16S rRNA primers MSfw: 5’-CCGGGTATCTAATCCGGTTC-3’ and MSrev: 5’- CTCCCAGGGTAGAGGTGAAA-3’.

The following thermal cycling parameters were used for amplification of DNA: 95°C for 10 minutes followed by 40 cycles of 15 seconds at 95°C, 30 seconds at 60°C, and 30 seconds at 72° C. A melting curve analysis was also performed to verify amplicon specificity.

### Short chain fatty acid (SCFA) measurement

SCFA concentrations were assessed in accordance with the method proposed by Weaver et al. [39], modified as follows. Stool (200 mg) were suspended in 1 ml of double distilled water, homogenized on vortex mixer and, after 30 min, centrifuged (15000 rpm) for 15 min at 10°C. Aliquots (500 μl) of supernatant were added with 200 μl 85% orthophosphoric acid, 200 μl 2% (v/v) sulphuric acid and 100 μl 2-ethyl-butyric acid (Sigma-Aldrich, Italy) 10 mM in HCOOH 12% as internal standard. SCFA were gently extracted for 1 min with 1 ml ethyl-ether/heptane (1:1 v/v) and centrifuged for 10 min at 3000 rpm. The aqueous phase was frozen and the organic layer was removed for analysis by a Varian 3400 CX gas liquid chromatograph equipped with a Varian 8200 CX auto sampler and a HP-FFAP fused-silica capillary column (30 m, 0.53 mm i.d. with a 1-mm film). Specific chromatography conditions were: gas carrier He with flow 15 ml/min; splitting 1:10 after 20 seconds injection; injection volume of 1μl. Injector and detector temperatures were 110 and 260°C, respectively. The initial oven temperature was 60°C and was increased by 10°C min-1 to 110° C and then by 5°C min-1 and held at 200 for 5 min. Quantification of the SCFA was obtained through calibration curves of acetic, propionic, *iso*-butyric, butyric and *iso*-valeric acid in concentrations between 0.25 mM and 10 mM (10 mM 2-ethyl-butyric acid as internal standard).

Serum SCFAs were determined according to the method proposed by Brighenti et al. [40] modified as follow. Briefly, 1 ml of serum was mixed with 100μl metaphosphoric acid (50%, v/v) and subsequently centrifuged at 22°C for 10 min. 500 μl of supernatant were added with 50μl 2-ethyl-butyric acid (Sigma-Aldrich, Italy) 10 mM in 2% (v/v) sulphuric acid, 200μl 10% (v/v) sulphuric acid and 1 ml of ethyl ether mixture. Samples were mixed for 2 min and centrifuged for 10 min. Samples were stored at -80°C for one hour, in order to quickly freeze the aqueous phase and facilitate the recovery of the organic phase containing volatile fatty acids. The extracts were stored at -20°C for the following gas-chromatography (GC) analysis.

Specific chromatography conditions were: gas carrier He with flow 22 ml/min; splitting 1:10 after 20 seconds injection; 2μl of injection volume; injector at 200°C and detector FID at 260°C. Quantification of the SCFA was obtained through calibration curves of acetic acid in concentrations between 0.05 mM and 0.5 mM.

### Statistical analysis

Statistical analysis was performed using the statistical software R (https://www.r-project.org/) and Graph Pad Prism (Graph Pad Software, La Jolla, CA). The statistical significance of factors affecting microbiota composition was evaluated using non-parametric permutational multivariate analysis of variance (perMANOVA) and significant clustering of groups was evaluated with analysis of similarities (ANOSIM). Ordination analysis was performed using redundancy analysis (RDA) in the vegan package [41] and alpha and beta diversity metrics were also calculated in the vegan package.

Statistical analysis of cohort-related characteristics comparing AN versus CTR, were performed using Student’s t-test for normally distributed samples and Wilcoxon test for non normally distributed samples, correlation analysis (Pearson and Spearman correlation coefficient) and linear regression models. Variables were expressed as mean ± standard deviation (SD), and for multiple comparisons p-values were adjusted with the False Discovery Rate method (FDR). A p-value less than or equal to 0.05 was considered statistically significant.

## Results

### Cohort description

Table 1 shows anthropometric, biochemical and hematological parameters statistically different in the two studied groups.

**Table 1:** Characteristics of the study population.

	AN (*n* = 15)	CTR (*n* = 15)	Reference range^§^
**Weight** (Kg)****	37.0±6.5	59.3±9.3	-
**BMI** (kg/m^2^) ****	13.9±2.1	22.1±2.6	-
**FM** (Kg) ****	1.6±1.1	14.4±7.0	-
**FFM** (kg) ***	35.2±5.9	44.9±3.7	-
**MM** (kg) ****	33.3±5.3	42. 6±3.5	-
**BM** (kcal/die) ****	1,088±137	1,554±732	-
**WBC count (**10^3^/μL)*	4.5±1.8	6.1±1.7	3.6–9.2
**CHE** (U/L)*	5,960±1388	6,886±1288	4,650–10,440
**Insulin** (μU/ml) ****	3.4±1.7	7.7±2.6	0–25
**ALT** (U/L) ***	44.5±18.3	25.4±7.7	9.0–52.0
**AST** (U/L) ***	35.3±11.2	22.1±4.2	14.0–36.0

Data are expressed as mean ± standard deviation. AN, anorexia nervosa; CTR, control group; BMI, body mass index; FM, fat mass; FFM, free fat mass; MM, muscle mass; BM, basal metabolism; WBC count, white blood cells count; CHE, cholinesterase; ALT, alanine aminotransferase; AST, aspartate aminotransferase.

**** *p* <0.0001

***p <0.001

*p ≤ 0.05.

^**§**^Reference range for biochemical parameters.

As expected, significant differences were observed in body mass index (BMI) at stool sample collection time, and body composition. Biochemical tests revealed in AN group higher values of ALT and AST.

Diet analysis confirmed the characteristic energy restriction in AN diet; compared with CTR group, AN subjects showed a lower dietary intakes of energy (1,195 Kcal/die ± 264 *vs* 1,481 Kcal/die ± 209, *p* **=** 0.009), total fats (33 g/die ± 11 *vs* 54 g/die ± 13 *p<*0.001) and carbohydrates (175 g/die ± 42 *vs* 204 g/die ± 27, *p* = 0.049). No significant differences were observed in fiber and protein intakes.

Psychopathological tests revealed significantly higher levels of depression, anxiety, and eating disorder psychopathology in AN group compared with controls (Table 2).

**Table 2:** Raw scores for psychopathological assessment.

		AN (*n* = 15)	CTR (*n* = 15)	*p*-value
**EDI-2**	**Drive for thinness**	11.4±7.4	0.9±2.4	<0.0001
	**Bulimia**	3.2±4.5	1.2±2.6	n.s.
	**Body dissatisfaction**	10.6±5.4	4.4±5.1	0.002
	**Effectiveness**	9±8.1	0.6±0.9	<0.0001
	**Perfectionism**	4.8±3.5	2±1.8	0.013
	**Interpersonal distrust**	5.2±4.7	0.8±1.2	0.005
	**Ineroceptive**	8.0±7.6	0.6±2.1	<0.001
	**Awarness**	7.5±4.8	2.4±2.2	0.001
	**Maturity fears**	6.8±5.3	1.8±1.1	0.003
	**Impulse regulation**	4.7±5.5	0.6±1.3	0.004
	**Social insecurity**	7.1±6.8	1.2±1.8	0.001
**SCL90**	**Somatization**	0.8±0.5	0.4±0.4	n.s.
	**Obsession-compulsion**	1.2±0.7	0.4±0.3	0.002
	**Interpersonal**	1.1±0.8	0.4±0.4	0.009
	**Depression**	1.6±1.0	0.4±0.3	<0.001
	**Anxiety**	1.2±0.8	0.4±0.3	0.004
	**Hostility**	0.9±0.8	0.3±0.4	0.020
	**Phobic anxiety**	0.6±0.8	0.1±0.2	0.003
	**Paranoid ideation**	1.1±0.8	0.4±0.5	0.021
	**Psychoticism**	0.7±0.5	0.1±0.2	<0.001
	**Sleeping**	1.7±1.2	0.4±0.71	0.001
	**Global index**	1.1±0.6	0.4±0.2	<0.001
**STAI-Y**	**State anxiety**	47.4±14.1	33.1±5.4	0.001
	**Trait anxiety**	53.6±11.1	34.1±5.4	<0.0001
**BDI**	**Total score**	20.4±11.9	2.5±2.6	<0.0001

Data are expressed means ± standard deviation. AN, anorexia nervosa; CTR, control group; n.s., not significant; EDI-2, Eating Disorder Index-2; SCL90, Symptoms Checklist-90 revised; STAI-Y, State Trait Anxiety Inventory, form Y; BDI, Beck Depression Inventory.

Extensive data description can be found in the supplementary S1 Table.

### Gut microbiota dysbiosis in anorexia nervosa subjects

Sequencing libraries had a median size of 165,200 sequences (min = 97,010; max = 339,500) and had a high coverage (median = 99.2% [min = 98.9%; max = 99.7%]). To avoid biases related to uneven library depth, sequencing libraries were subsampled to a number of reads smaller than the smallest library (97,000 reads).

Bacterial composition within each sample (α-diversity) and between CTR and AN groups (β-diversity) was measured by using operational taxonomic unit (OTU)-based methods. All metrics estimates were not statistically significant.

**Fig 1:**
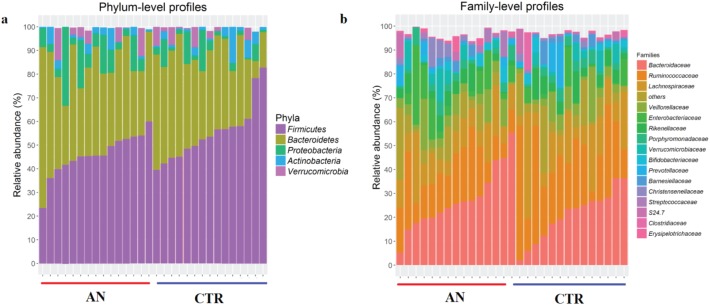
Relative abundance of common microbial taxa. Bar charts showing the relative abundance of the most represented microbial taxa, defined as having a mean relative abundance of >1%, in stool samples of anorexia nervosa (AN, n = 15) and control (CTR, n = 15) groups. Phylum-level (**a**) and family-level (**b**) taxon profiles are shown.

Gut microbiota composition at phylum and family level is reported in Fig 1 for the AN and CTR groups.

At phylum level, the predominant bacterial taxa were *Firmicutes* and *Bacteroidetes* followed by *Proteobacteria*, *Actinobacteria* and *Verrucomicrobia* (Fig 1A). The most abundant families were *Bacteroidaceae*, *Ruminococcaceae*, *Lachnospiracea*, *Veillonellaceae*, *Enterobacteriaceae*, *Rikenellaceae*, *Porphyromonadaceae*, *Verrumicrobiaceae*, *Bifidobacteriaceae*, and *Prevotellaceae*, (Fig 1B). The most abundant genera were *Bacteroides*, *Ruminococcus*, *Dialister*, *Faecalibacterium*, *Akkermansia*, *Parabacteroides*, *Bifidobacterium*, and *Prevotella* (S1 Fig).

Significant differences in taxa between AN and CTR are reported in Table 3.

**Table 3:** Taxa significantly increased (+) or decreased (-) in abundance in anorexia nervosa subjects.

Taxonomic level	Taxa	Relative abundance		AN	*p*-value
		CTR	AN		
Phylum	*Firmicutes* *Proteobacteria*	55.2±12.1 4.7±5.3	45.9±8.8 8.9±8.9	- +	0.041 0.031
Family	*Ruminococcaceae*	25.6±13.6	16.6±8.0	-	0.033
	*Enterobacteriaceae*	2.7±4.9	7.6±9.2	+	0.047
Genus	*Ruminococcus*	4.8±3.8	2.2±2.4	-	0.019
	*Roseburia*	2.3±2.2	0.8±0.9	-	0.037
	*Clostridium*	1.4±3.6	0.2±0.2	-	0.031

Data are expressed means ± standard deviation. AN, anorexia nervosa; CTR, control group.

**Fig 2:**
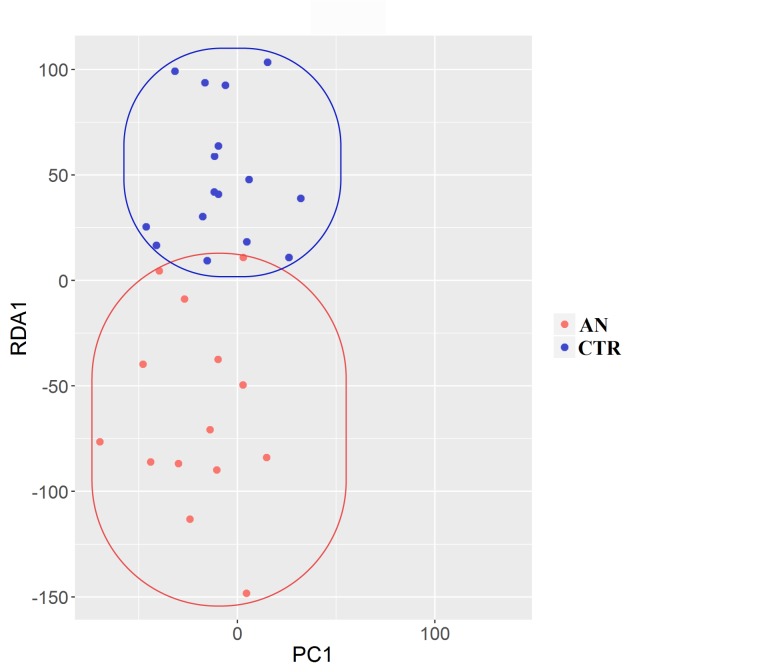
Redundancy analysis (RDA) of gut microbiota. RDA shows a separation between anorexia nervosa (AN, red dots) and control (CTR, blue dots) subjects.

The composition of the intestinal microbiota was significantly affected by anorexia status at every taxonomic level, as determined by non-parametric multivariate analysis of variance (perMANOVA; *p*<0.05 for all levels). Redundancy analysis (RDA) showed that samples were distinctly grouped (Fig 2).

This grouping was confirmed at OTUs level by the analysis of similarity (ANOSIM) test, which evaluates significance of sample grouping (*p* = 0.03).

Moreover, a significant increase of Gram-negative bacteria was observed in AN participants compared with CTR (*p* = 0.03).

Archeabacteria, with the main genus *Methanobrevibacter*, were detected in 30% of AN and CTR subjects. Quantification of *Methanobrevibacter smithii* using real-time PCR has shown a higher average of genome copy number of the archeon in AN group (*p* = 0.0259).

### Fecal and systemic microbial metabolites

Fecal concentrations of total SCFAs, butyrate and propionate were significantly (p<0.05) decreased in AN samples, while acetate, *iso*-valerate and *iso*-butyrate were not different between groups (Table 4).

**Table 4:** Fecal SCFA concentrations in anorexia nervosa subjects and healthy control group.

	Fecal SCFA (mg/g feces)		
	AN	CTR	*p*-value
Total SCFAs	4.7±2.1	6.8±1.6	0.041
Acetate	2.7±1.5	3.8±1.1	n.s.
Butyrate	0.7±0.4	1.1±0.5	0.045
Propionate	0.8±0.4	1.1±0.3	0.028
*Iso*-valerate	0.4±0.2	0.5±0.2	n.s.
*Iso*-butyrate	0.2±0.1	0.2±0.1	n.s.

Data are means ± standard deviation. AN, anorexia nervosa; CTR, control group; n.s., not significant

Acetate was the only SCFA detected in serum, with no significant differences in the two experimental groups (AN; 0.037 μM ± 0.02 (mean ±SD), CTR; 0.042 μM ±0.02). No significant relationship was observed between systemic and fecal acetate.

### Complex interplay between psychopathology tests, clinical status and microbiota profiles

Correlation tests were carried out in order to find possible associations between gut microbiota composition and cohort characteristics. The correlation analysis performed at OTUs level revealed two OTUs related to insulin and BMI, respectively. OTU 3485 (100% sequence similarity with Accession Number NR_042007.1 *Roseburia inulinivorans*) was positively correlated with insulin values (*p* = 0.0397), while the OTU 5247 (99% sequence similarity with Accession Number NR_112945.1 *Bacteroides uniformis*) was negatively related with BMI (*p* = 0.0194).

Other biochemical parameters were not significantly associated with gut microbiota composition.

PerMANOVA analysis confirmed the significant correlations between gut microbiota, insulin and BMI; insulin correlated with gut communities at OTUs level (*p* = 0.002), and BMI at every taxonomic level (p < 0.05).

BMI was also positively associated with SCFA concentration (total SCFAs, *p* = 0.0125; butyrate, *p* = 0.0035; propionate, *p* = 0.0137; and *iso*-Butyrate, *p* = 0.0282).

**Fig 3:**
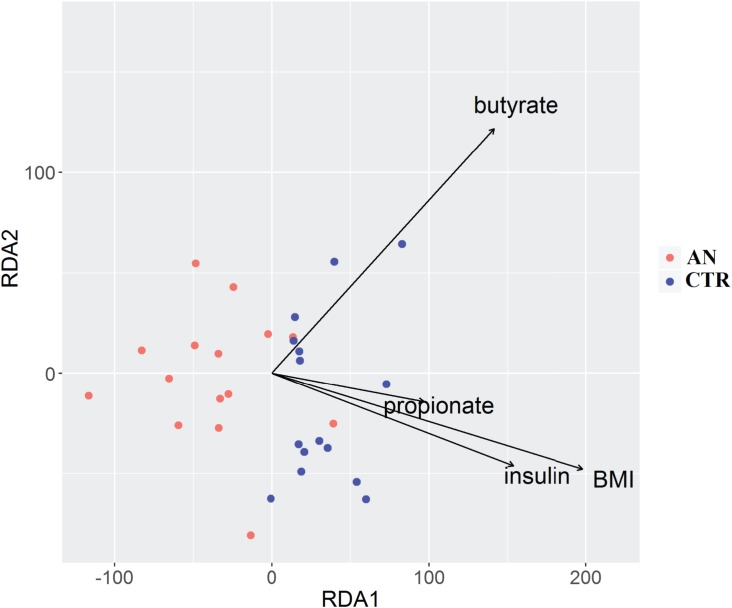
Distance-based redundancy analysis (db-RDA). Db-RDA plot shows correlations between gut microbiota composition and body mass index (BMI), insulin, propionate and butyrate. Only significant variables are represented (p>0.05, Permanova analysis). Arrows in the db-RDA biplot denote the magnitudes and directions of the variable effects. Controls are represented by blue dots and anorexic subjects by red dots.

Association between BMI, insulin and propionate was revealed by RDA analysis (Fig 3).

In a multivariate analysis, we found BMI to be negatively associated (p<0.05) with the following psychopathologic parameters: obsession-compulsion, depression, and anxiety (SCL-90 and BDI inventory).

We then investigated possible correlation between microbiome and psychological traits.

Statistical analysis revealed a negative correlation only between BDI depression score and *Clostridium* spp. (*p* = 0.0089), whereas fecal butyrate concentration inversely correlated with depression (*p* = 0.0379) and anxiety (*p* = 0.0206) scores.

## Discussion

Our study described clinical and anthropometric parameters, psychological status, microbiota and SCFA profiles in anorexia nervosa subjects, integrating the current literature on gut dysbiosis in AN [1,28,29,42].

Anorexia status was characterized by an altered intestinal microbiota composition, with unbalanced Gram positive/Gram negative relative abundance. The microbiota of AN subjects was enriched in *Bacteroidetes* and depleted in *Firmicutes*, thus showing an opposite trend compared with what described in obese subjects [12,20]. In particular, the relative abundance of the carbohydrate-fermenter *Ruminococcus*, *Roseburia* and *Clostridium* genera, belonging to *Firmicutes*, was decreased in AN. This is in agreement with previous data [1,29], and supports the significantly lower fecal butyrate concetration assessed in AN subjects. *Roseburia* spp., such as *R*. *faecis*, *R*. *inulinivorans*, *R*. *intestinalis*, and *R*. *hominis*, are important butyrate-producing species. *Roseburia intestinalis* is a mucosa-associated species that increases the bioavailability of butyrate as energy source for colonocytes. *R*. *intestinalis* has been suggested to be also a polyamine producer, thus essential for the maintenance and function of gastrointestinal epithelium. A reduced polyamine production could alter the structure of the epithelial layer, affecting the survival of adherent microbes that in turn could have a significant effect on energy harvest from food [43].

Decreased *Roseburia* spp. abundance has been also found in patients with infammatory bowel diseases [44]. Altered intestinal permeability [45] and low-grade inflammation [46] has been also shown in animal models of AN, suggesting a putative role in the pathophysiology of AN.

Both pro-inflammatory cytokines, mainly IL-1β and TNF-α [47], and mucosal microbes could affect feeding behavior and satiety [48]; in particular, microbially derived SCFAs can promote the release by enteroendocrine cells of peptide YY (PYY) and glucagon-like peptide (GLP) that in turn regulate appetite and insulin secretion.

The Gram-negative *Proteobacteria*, were also enriched in AN microbial community, and *Enterobacteriaceae* were overrepresented compared with control subjects. *Enterbacteriaceae* have been associated to gut inflammation that in turn favors bacterial translocation, promoting systemic inflammation [49].

A recent report suggested a new role for *Enterobacteriaceae* in eating disorders; this family, and in particular *Escherichia coli* species, can produce, amongst other metabolites, an anorexigenic and anxiogenic protein, the caseinolyitic protease b (ClpB) [50]. ClpB seems to interfere with α-melanocyte-stimulating hormone involved in satiety and anxiety signaling [51]. Breton and colleagues found an association between eating disorders and ClpB [50]. In agreement with this observation, the increased abundance of Gram negative bacteria might be linked to a higher production of neuropeptide ClpB, which could mediate in the communication between the gut-brain axis in AN subjects.

Moreover, AN microbial communities were enriched in *Methanobrevibacter smithii*. This *Archea* species is able to transform hydrogen in methane, and allows extracting extra calories from nutrients [28]. However, *M*.*smithii* has been found in the microbiota of patients suffering of obesity, non-alcoholic fatty liver disease, and cirrhosis [52]. Liver dysfunction is a common feature in AN and an increase in liver enzymes has been previously described [53,54] and confirmed in this study.

Changes in the abundance of microbial communities lead to changes in the quantity/quality of microbial metabolites. Indeed, reduction in *Firmicutes* is in line with the lower fecal butyrate concentration in AN group. Interestingly, butyrate was negatively correlated with depression and anxiety. Sodium butyrate has been demonstrated to elicit antidepressant effects on murine brain. When injected systemically, sodium butyrate induced a short-lasting, transient acetylation of histones in frontal cortex and hippocampus, in conjunction with dynamic changes in expression of brain-derived neurotrophic factor (BDNF), thereby resulting in an antidepressant-like behavioral response [55].

Faecal propionate was also significantly reduced in AN subjects. A depletion in *Roseburia* species, and in particular of *Roseburia inulinivorans*, could result in a decrease production of propionate [56]. *R*. *inulinivorans* produces propionate from fucose via propanediol, and butyrate when grown in the presence of glucose [57]. Interestingly, our data showed a significant direct correlation between this species and insulin concentration, and insulin levels in AN group were significantly lower than healthy controls. This “profile” suggests a possible link between *Roseburia* species and insulin metabolism. A reduced insulin is a phenomenon well documented in literature [58] that helps AN subjects to preserve euglycemia.

Propionate has beneficial effects on β-cell function, potentiating glucose-stimulated insulin release and maintaining β-cell mass through inhibition of apoptosis [59]. On the other hand, propionate has been shown to inhibit adipogenesis [60], and the link might be represented by reduced insulin levels.

Other biochemical parameters were found altered in AN group; white blood cell count, and cholinesterase level were reduced, whereas transaminase were increased. These alterations have been already reported in literature [3,61] and represent both a clinical adaptation and a marker of the effects of starvation and malnutrition.

Anorexic patients, as expected, showed reduced BMI, fat mass, and free fat mass if compared with healthy subjects. BMI is considered a severity index in AN patients, especially to define enduring AN [62]. Despite nutritional and psychological support, all enrolled subjects failed to recover BMI or stabilize weight gain, leading to a fluctuation in BMI as previously reported [63].

BMI resulted a good predictive value for dysbiosis level. The correlation analysis revealed that *Bacteroides uniformis* was negatively correlated with BMI, in agreement with our recent study on obese children in which members of the *Bacteroidetes* phylum were generally good predictors of BMI [20].

BMI was also inversely correlated with all psychological tests, and in particular with obsession-compulsion, depression, and anxiety. Longitudinal studies on AN cohorts suggested that weight gain during renourishment, leads to improvements of psychological symptoms [6].

In this study, we observed that fecal butyrate concentration and *Clostridium* spp. negatively correlate with anxiety and depression scores. This result is consistent with Bailey et al. [64] who demonstrated that exposure of mice to a social disruption stressor results in a significant decreases in *Clostridium* spp. abundance.

In order to understand whether short-chain fatty acids could exert a direct effect on gut-brain axis by entering the systemic circulation, we evaluated their concentrations in plasma. Acetate was the only metabolite found in our samples, whereas propionate and butyrate were undetectable. Indeed, acetate is the most abundant metabolite produced and released systemically. In healthy subjects the molar ratio of acetate:propionate:butyrate in peripheral blood is approximately 80:10:10, representing 1/1000th of the concentration present in the large intestine [65].

Systemic acetate can cross the blood–brain barrier and has been demonstrated to directly suppress appetite through central hypothalamic mechanisms in mice [66]; however no significant differences were observed in plasma acetate concentration between the two studied groups.

It should be considered that SCFAs could affect the gut-brain axis also through a modulation of the enteric nervous systems, by stimulating gut hormones and cytokine release or directly via afferent neural pathways [67].

In conclusion, in the present study a significant dysbiosis was observed in AN patients, with specific changes in microbial genera that might participate in AN pathophysiology. More studies will be needed for dissecting if the restricted diet consumed by AN patients, starving the microbiota, is responsible for the observed alterations.

The finding of reduced fecal butyrate and butyrate-producing genera, such as *Roseburia* spp., indicates microbial modulation as a possible tool to implement current AN treatment. Indeed, it has been shown both in humans and in mice that probiotic supplementation with lactobacilli and bifidobacteria, by stimulating a cross-feeding mechanism, increases *Roseburia* abundance and butyrate production [68]. Moreover, pre- and probiotic administration could represent a feasible intervention for AN patients because of its extremely low calories or calorie-free content, improving recovery rates.

Taken together our data highlight the close relationship between microbial community, peculiar diet and psychiatric disorders, opening important questions to be addressed for ascertaining causes/consequences of observed dysbiosis in AN.

## Supporting information

S1 Table Cohort description (demographic, anthropometric, biochemical, and psychological data), diet and short-chain fatty acids concentrations. (XLSX)

S1 Fig Relative abundance of most represented microbial genera. Bar charts showing the relative abundance of the most represented genera, defined as having a mean relative abundance of >1%, in stool samples of anorexia nervosa (AN, n = 15) and control (CTR, n = 15) groups.
(EPS)

## Abbreviations

BMI: body mass index
FM: fat mass
FFM: free fat mass
MM: muscle mass
BM: basal metabolism
WBC count: white blood cells count
CHE: cholinesterase
ALT: alanine aminotransferase
AST: aspartate aminotransferase
EDI-2: Eating Disorder Index-2
SCL90: Symptoms Checklist-90 revised
STAI-Y: State Trait Anxiety Inventory, form Y
BDI: Beck Depression Inventory
